# Causes and risk factors for deaths in young infants in South Asia: the ANISA prospective population-based observational cohort study

**DOI:** 10.1136/bmjgh-2024-018433

**Published:** 2025-11-03

**Authors:** Gary L Darmstadt, Safa Abdalla, Mohammad Shahidul Islam, Shams El Arifeen, Melissa L. Arvay, Abdullah H Baqui, Zulfiqar A Bhutta, Anuradha Bose, Nicholas E Connor, Belal Hossain, Rita Isaac, Arif Mahmud, Dipak K. Mitra, Luke C Mullany, Imran Nisar, Kalpana Panigrahi, Pinaki Panigrahi, Qazi Sadeq-ur Rahman, Senjuti Saha, Sajid B. Soofi, Nardos Solomon, Mathuram Santosham, Stephanie J Schrag, Shamim A. Qazi, Samir K Saha

**Affiliations:** 1 Department of Pediatrics, Stanford University School of Medicine, Stanford, California, USA; 2 Child Health Research Foundation, Dhaka, Bangladesh; 3 Maternal and Child Health Division, International Centre for Diarrhoeal Disease Research Bangladesh (icddrb), Dhaka, Bangladesh; 4 Centers for Disease Control and Prevention, Atlanta, Georgia, USA; 5 Department of International Health, Johns Hopkins University Bloomberg School of Public Health, Baltimore, Maryland, USA; 6 Centre for Global Child Health, The Hospital for Sick Children, Toronto, Ontario, Canada; 7 Institute for Global Health and Development, The Aga Khan University, Karachi, Pakistan; 8 Christian Medical College and Hospital Vellore, Vellore, India; 9 Department of Research Services, University of Oxford, Oxford, UK; 10 Public Health, North South University, Dhaka, Dhaka District, Bangladesh; 11 Department of Paediatrics and Child Health, Aga Khan University, Karrachi, Pakistan; 12 AIPH University, Bhubaneswar, India; 13 Department of Pediatrics, Georgetown University Medical Center, Washington, District of Columbia, USA; 14 Center of Excellence in Women & Child Health, Aga Khan University, Karachi, Pakistan; 15 Independent Consultant Paediatrician, Geneva, Switzerland

**Keywords:** Global Health, Child health, Epidemiology, Paediatrics, Public Health

## Abstract

**Introduction:**

Strategies for reducing infant mortality require accurate, local, population-level data. We conducted a population-based observational study in three countries in South Asia to describe risk factors, causes and rates of mortality in young infants.

**Methods:**

Pregnancies, births and pregnancy outcomes were determined through household surveillance, and cause of deaths was ascertained by verbal autopsy. Cox regression was used to identify risk factors for deaths during days 0–<3, 3–<7 and 7–<60.

**Results:**

Among 73 622 pregnancy outcomes, 4638 deaths were identified, including 1669 stillbirths (36.0%), 1347 (29.0%) deaths among non-registered liveborn infants who died before the first home visit by community health workers (CHWs), and 1622 (35.0%) deaths that occurred during days 0–<60 among liveborn registered infants. Most deaths among liveborn infants (59.3%, 1757 of 2965) took place within 3 days of birth. The most common causes of death over the young infant period were infections/sepsis (32.5%, n=963 of 2,965), birth asphyxia (29.0%, n=859) and preterm birth/low birth weight (14.1%, n=418). Risk factors for mortality included early morbidity (need for resuscitation, intrapartum infection/antibiotics, multiple gestation, congenital anomalies), environmental factors (smoke exposure, maternal betel chewing) and poor maternal access to quality care (history of a prior neonatal death, lack of care seeking for labour complications). Protective factors included biology (female sex, higher birth weight), essential newborn care (immediate breastfeeding, clean cord care) and access to quality maternal and newborn care (antenatal care, facility birth, skilled birth attendant, maternal education, household wealth).

**Conclusions:**

Our population-based data highlight the importance of addressing deaths due to birth asphyxia and infections, while recognising that the relative burden of deaths due to preterm birth and congenital anomalies is increasing globally. Access to quality community-based and facility-based maternal and newborn care is critical to efforts to reduce mortality in young infants in high-mortality settings such as rural South Asia.

**WHAT IS ALREADY KNOWN ON THIS TOPIC**

- Data from low-resource settings on cause-of-death among young infants 0–<60 days of age is scarce; thus, estimates rely on modelling of global data, which identifies complications of preterm birth as the top cause.

**WHAT THIS STUDY ADDS**

- Intensive prospective household surveillance for pregnancies and births, and postnatal household visits of over 70 000 infants by community health workers in five sites in Bangladesh, India and Pakistan revealed high rates of mortality in the first 2 months after birth: 31.5 stillbirths per 1000 total births, 43.0 young infant deaths per 1000 live births and 60.6 perinatal deaths per 1000 births.
- Young infant deaths occurred early (nearly 60% within 3 days of birth), primarily due to infection/sepsis (32.5%) and birth asphyxia (29.0%), followed by preterm/low birth weight (14.1%).

**HOW THIS STUDY MIGHT AFFECT RESEARCH, PRACTICE OR POLICY**

- While neonatal health programming and research currently emphasise facility-based care for small and sick or small vulnerable newborns, programmes must continue to focus on addressing birth asphyxia and infections as primary causes of death at the population level in low-resource settings in South Asia, and also recognise that preventing and managing preterm birth and congenital anomalies also require concerted attention.

## Introduction

 Development of research priorities, country policies and programmes aimed to reduce neonatal and child mortality relies on accurate, timely, population-representative data. However, these data are relatively scarce in low-resource settings,^1 2^ and as a result, globally available data are modelled to estimate rates and cause distributions of deaths.^3^,^7^ These analyses suggest that, in general, proportions of newborn deaths due to complications of preterm birth and congenital anomalies have increased over the past two decades, while deaths due to intrapartum-related events (birth asphyxia) and serious infections have decreased. The primary cause of death in neonates and under-five children based on modelled estimates is complications of preterm birth globally, regionally in South Asia and specifically in Bangladesh, India and Pakistan.^4 8^ Funding and global research and programme priorities currently place emphasis on quality of care, particularly at birth and in the early postnatal period. Care of small and/or sick newborns is also emphasised, in recognition that more than half of newborn deaths are estimated to occur in small vulnerable infants (preterm, small-for-gestational age and/or low birth weight (LBW)) who require care in a health facility, and approximately three-fourths of these deaths occur in preterm infants.^69^,^13^

The Alliance for Maternal and Newborn Health Improvement (AMANHI) mortality study group published data from 2012 to 2016 on the burden, timing and causes of maternal deaths, stillbirths and neonatal deaths determined by verbal autopsy in population-based surveillance sites in South Asia and sub-Saharan Africa. They showed that the rate of neonatal mortality in South Asia is two-fold that of estimates from sub-Saharan Africa and that proportions of deaths due to perinatal asphyxia and severe neonatal infections are about twice that of preterm birth, differing from global estimates.^814^,^16^

The Aetiology of Neonatal Infection in South Asia (ANISA) study was a prospective, community-based observational cohort study implemented in five sites in India, Bangladesh and Pakistan from 2011 to 2015 to identify the aetiology of neonatal infections at population level.^17 18^ The study was designed with an emphasis on illuminating the causes of bacterial and viral infections with particular emphasis on capturing those of early onset in the first week after birth, given the scarcity of these data. Particular attention was given to rigorous surveillance for pregnancies, births and pregnancy outcomes. Community health workers (CHWs) continued to follow infants through regular home visits throughout the young infant period (the first 60 days after birth) to identify signs of illness so that samples could be collected for identification of infections and to ascertain vital status.^19^ We used this infrastructure to address the following primary research questions: what are population-based rates of stillbirths and mortality among young infants in South Asia, and what are the primary causes and risk factors for deaths?

## Methods

### Data

We used data from the ANISA prospective community-based observational cohort study conducted in 2011–2015 in Sylhet, Bangladesh, Vellore and Odisha, India, and Karachi and Matiari, Pakistan, described in detail elsewhere^17 18 20^ and reported according to Strengthening the Reporting of Observational Studies in Epidemiology (STROBE) guidelines for cohort studies, with reference to STROBE for Newborn Infection.^21 22^ All the study sites had recently completed a population census, including the identification of all households. Bimonthly surveillance of all households in the study communities was conducted to identify married women of reproductive age (13–49 years), who were then visited every 2 months to ascertain pregnancy status based on the lapse of >2 months since the last menstrual period. Pregnant women were consented and enrolled and entered into a birth notification system. Beginning at the 37th week of pregnancy, families were contacted every other day to inquire about the delivery status. CHWs collected information related to the household, socioeconomic status, reproductive history and nutritional status at enrolment and information on pregnancy outcome, antenatal care, labour and birth history at the initial visit after childbirth. Each pregnancy outcome was recorded as soon as possible. Newborns identified within 7 days of birth were registered and followed up by CHWs during up to ten scheduled home visits (at least one per week) from birth through 60 completed days.^18^ Infants identified by CHWs after 7 days post birth or who died prior to registration were unregistered and were followed only to identify deaths and conduct verbal autopsies. At each postnatal visit of registered infants, CHWs assessed infants for signs of possible serious bacterial infections (pSBI) and vital status and referred infants with signs of infection for detection of aetiology and treatment as reported previously.^17 18^

#### Verbal autopsy methodology

In the event of stillbirths (death after 28 weeks of pregnancy, but before or during birth) or infant deaths identified during community-based infection surveillance, for both registered and unregistered infants, CHWs referred the cases to a supervisory member of the verbal autopsy team. A verbal autopsy team member made a home visit to conduct a verbal autopsy with the mother or the next closest guardian of the infant at the time of death if she was deceased. On average, the verbal autopsy interview was conducted 12 months after the death—an acceptable recall period^23^—and lasted approximately 1 hour. The verbal autopsy interview was designed to collect data for ascertainment of cause of death based on caregiver observations regarding the circumstances preceding and leading to the death of their infant.^24^,^29^ The verbal autopsy instrument used was adapted for our sites based on those developed by the AMANHI mortality study group using WHO verbal autopsy standard tools^30 31^ and included three sections: a narrative of the circumstances leading to death and timing of death, closed-ended questions and review of health records. Master trainers from all sites were trained in verbal autopsy procedures during two training workshops at WHO in Geneva. The master trainers then trained verbal autopsy supervisors and data collectors at the sites. For quality assurance, site supervisors conducted interviews and provided feedback on a random selection of 5% of the verbal autopsies conducted by the data collectors.

Physician verbal autopsy coders were trained, including in principles of the International Classification of Diseases-10, through an accreditation process overseen by WHO.^31^,^33^ The condition that occurred earliest and started the chain of events that resulted in death was considered the cause of the death. Two trained, accredited physicians independently coded each autopsy report and assigned a single cause of death; a third physician reviewed the verbal autopsy if agreement was not reached and made a final determination.

### Analysis

We merged the verbal autopsy study data with data collected from the mother about sociodemographics, pregnancy, delivery and postnatal care. We carried out descriptive analysis for the frequency of deaths in the cohorts during the first 60 days after birth by cause in both non-registered babies and registered babies stratified by age group (0–<3 days, 3–<7 days, 7–<60 days). Stillbirths were excluded from analyses. Cumulative mortality plots were generated overall and by registration status.

We selected four continuous variables and 35 categorical variables as potential risk factors for mortality; categorical variables were recoded where applicable to generate the categories reported (online supplemental table S1). A wealth index was constructed from household assets and characteristics using principal components analysis. We analysed the distribution of all potential risk factors and sought to find those which were associated with mortality due to each of four main causes of death identified by verbal autopsy (birth asphyxia (comparable to intrapartum-related events or neonatal encephalopathy), infection/sepsis (comparable to serious infections), congenital anomalies (sometimes also referred to as birth defects) and preterm birth/LBW (encompassing complications of preterm birth)) with more than five deaths in each of three time-frames (0–<3 days, 3–<7 days and 7–<60 days). Analyses of risk factors for congenital anomalies were excluded due to too few cases in each age group. We first used univariable Cox regression to model the association of mortality with each of the 39 variables. Factors with a univariable association with mortality at an alpha cut-off of <0.25—a widely accepted cut-off for this initial step in the analysis—were then entered into multivariable regression with backward selection, retaining only those factors that had a significant association at alpha cut-off of <0.05. Univariable associations of the selected variables are also reported along with the final step of the multivariable analysis. For mortality from preterm birth/LBW, we constrained the analysis to examine risk factors for mortality among infants who had been identified as having those conditions. All analyses used SAS V.9.4 and statistical significance was determined with an alpha cut-off of <0.05.

### Patient and public involvement

Patients and the public were not involved in the design, conduct, reporting or dissemination of this research.

## Results

### Causes of death

#### Study sample

**Figure 1:**
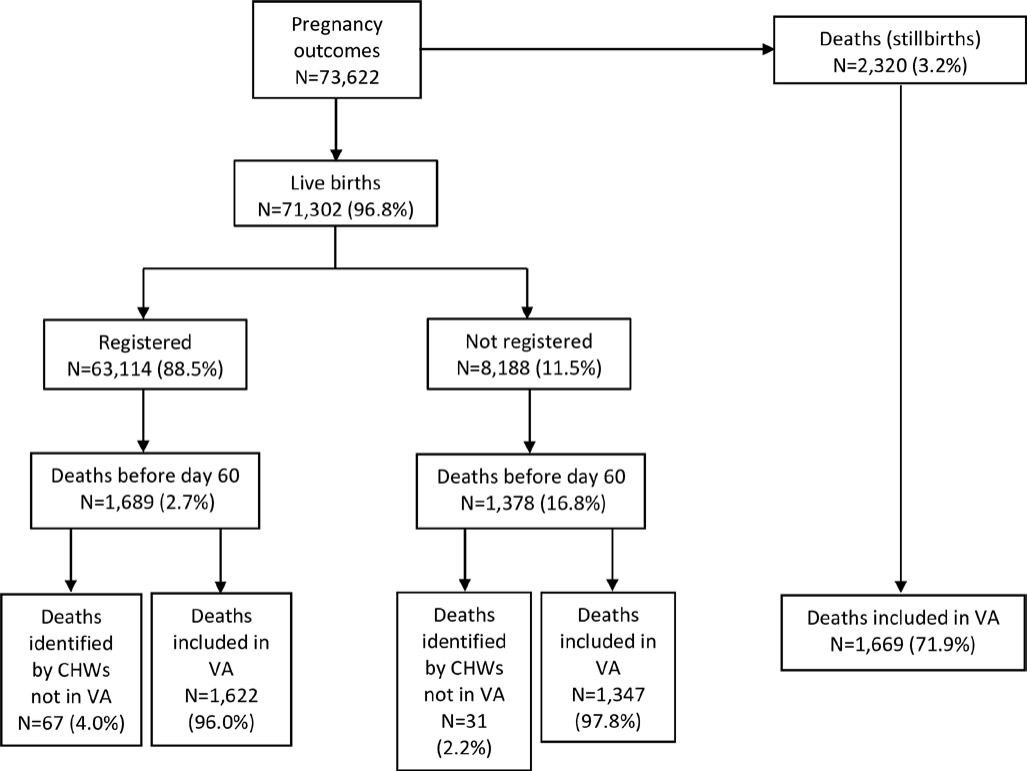
Deaths reported in the Aetiology of Neonatal Infection in South Asia verbal autopsy (VA) database by birth outcome and registration status. CHW, community health worker.

The ANISA study database included 73 622 pregnancy outcomes for women enrolled in the study, including 2320 stillbirths; among 71 302 liveborn infants there were 8188 unregistered infants and 63 114 registered infants who were enrolled and followed in the main ANISA study on aetiology of infection (figure 1).^16^ Reasons for non-registration were failure to reach the infant within the first 7 days after birth (n=6690, 81.7%), death of the infant before registration was completed (n=1377, 16.8%), migration out of the study area (n=111, 1.4%) and refusal to provide information for registration (n=10, 0.1%).

A total of 5387 deaths were identified. The overall stillbirth rate was 31.5 per 1000 total births (2320 of 73 622) (figure 1). The overall young infant mortality rate (days 0–<60 days) among unregistered and registered infants was 43.0 per 1000 live births (3067 of 71 302). The rate of perinatal mortality, encompassing stillbirths plus deaths in the first week after birth, was 60.6 per 1000 births (4459 deaths (2320 stillbirths, 257 unregistered infant deaths during days 0–<7, 882 registered infant deaths during days 0–<7) of 73 618 infants).

Overall, the verbal autopsy was administered to 86.1% (4638 of 5387) of all deaths identified: 97.8% (1347 of 1378) of unregistered infants, 96.0% (1622 of 1689) of registered infants and 71.9% (1669 of 2320) of stillbirths (figure 1). Reasons for not completing verbal autopsy included the family being not approached (70.3%, 527 of 749), having out-migrated (14.8%, 111 of 749), refusing (7.6%, 57 of 749), being absent (3.9%, 29 of 749) or others (3.3%, 25 of 749). There were four registered liveborn infants for whom the age and cause of death was not specified.

#### Cumulative causes of deaths for unregistered and registered infants

Among all young infants for whom data on age and cause of death was reported (n=2965), the most common causes of death were infection/sepsis (n=963, 32.5%) and birth asphyxia (n=859, 29.0%) followed by preterm/LBW (n=418, 14.1%) (table 1). A cause could not be determined for 17.0% (505 of 2965) of young infant deaths.

**Table 1:** Age distribution of causes of death of young infants in the Aetiology of Neonatal Infection in South Asia study, overall and stratified by registration status

A. All infants (unregistered and registered) (n=71 200)*				
Cause of death	0–<3 days	3–<7 days	7–<60 days	Total
Birth asphyxia	758 (43.1%)	58 (15.2%)	43 (5.2%)	859 (29.0%)
Infection/sepsis	349 (19.9%)	211 (55.2%)	403 (48.8%)	963 (32.5%)
Congenital anomalies	44 (2.5%)	6 (1.6%)	29 (3.5%)	79 (2.7%)
Diarrhoea	1 (0.1%)	0 (0%)	18 (2.2%)	19 (0.6%)
Preterm birth/low birth weight	332 (18.9%)	40 (10.5%)	46 (5.6%)	418 (14.1%)
Other causes†	67 (3.8%)	20 (5.2%)	35 (4.2%)	122 (4.1%)
Unknown/unspecified/undetermined	206 (11.7%)	47 (12.3%)	252 (30.5%)	505 (17.0%)
Total	1757 (100%)	382 (100%)	826 (100%)	2965 (100%)

B. Unregistered infants (n=8157)				
Cause of death	0–<3 days	3–<7 days	7–<60 days	Total
Birth asphyxia	549 (47.7%)	16 (15.1%)	5 (5.6%)	570 (42.3%)
Infection/sepsis	137 (11.9%)	54 (50.9%)	33 (36.7%)	224 (16.6%)
Congenital anomalies	36 (3.1%)	0 (0%)	2 (2.2%)	38 (2.8%)
Diarrhoea	1 (0.1%)	0 (0%)	0 (0%)	1 (0.1%)
Preterm birth/low birth weight	239 (20.8%)	14 (13.2%)	18 (20.0%)	271 (20.1%)
Other causes‡	43 (3.7%)	4 (3.8%)	2 (2.2%)	49 (3.6%)
Unknown/unspecified/undetermined	146 (12.7%)	18 (17.0%)	30 (33.3%)	194 (14.4%)
Total	1151 (100%)	106 (100%)	90 (100%)	1347 (100%)

C. Registered infants (n=63 047)*				
Cause of death	0–<3 days	3–<7 days	7–<60 days	Total
Birth asphyxia	209 (34.5%)	42 (15.2%)	38 (5.2%)	289 (17.9%)
Infection/sepsis	212 (35.0%)	157 (56.9%)	370 (50.3%)	739 (45.7%)
Congenital anomalies	8 (1.3%)	6 (2.2%)	27 (3.7%)	41 (2.5%)
Diarrhoea	0 (0%)	0 (0%)	18 (2.5%)	18 (1.1%)
Preterm birth/low birth weight	93 (15.4%)	26 (9.4%)	28 (3.8%)	147 (9.1%)
Other causes§	24 (4.0%)	16 (5.8%)	33 (4.5%)	73 (4.5%)
Unknown/unspecified/undetermined	60 (9.9%)	29 (10.5%)	222 (30.2%)	311 (19.2%)
Total	606 (100%)	276 (100%)	736 (100%)	1618 (100%)

* Four registered infants for whom age at death was not specified, and cause of death was unknown/unspecified/undetermined, are excluded.

† Respiratory distress (13 deaths) + pregnancy-induced hypertension (13 deaths) + obstetric complications (21 deaths) + miscellaneous (75 deaths)

‡ Respiratory distress (four deaths) + pregnancy-induced hypertension (12 deaths) + obstetric complications (14 deaths) + miscellaneous (19 deaths)

§ Respiratory distress (nine deaths) + pregnancy-induced hypertension (one death) + obstetric complications (seven deaths) + miscellaneous (56 deaths)

The majority of young infant deaths (59.3%, 1757 of 2965) took place within 3 days of birth (table 1, online supplemental figure S2A). The most common cause of death in the first 3 days was birth asphyxia (n=758, 43.1%) followed by infection/sepsis (n=349, 19.9%) and preterm/LBW (n=332, 18.9%) (table 1). Infection/sepsis was the most common cause of death during days 3–<7 (56.9%, 157 of 276) and 7–<60 (50.3%, 370 of 736).

Among deaths attributed to birth asphyxia and preterm/LBW, a preponderance occurred in the first 3 days (88.2% of young infant deaths due to birth asphyxia, 758 of 859; 79.4% of young infant deaths due to preterm/LBW, 332 of 418) (table 1, online supplemental figure S2A), whereas deaths due to infection/sepsis were distributed more evenly over the young infant period, with about one-third (36.2%, 349 of 963) of deaths occurring in the first 3 days and 58.2% (560 of 963) in the first week. Among deaths from congenital anomalies, about half (55.7%, 44 of 79) occurred in the first 3 days and nearly two-thirds (63.3%, 50 of 79) took place in the first week.

#### Causes of deaths among unregistered infants

The overall mortality rate in the young infant period among unregistered infants in the verbal autopsy study was 165 per 1000 live births (1347 of 8157; 31 deaths identified by CHWs among unregistered infants were not included in the verbal autopsy study) (figure 1; table 1). Cause of death could not be determined overall for 14.4% of non-registered infants (194 of 1347). The vast majority (85.4%, 1151 of 1347) of deaths in unregistered infants occurred in the first 3 days after birth (before they could be registered) (table 1, online supplemental figure S2B). Birth asphyxia (47.7%, 549 of 1151) was the most common cause of death in the age group 0–<3 days, whereas infection/sepsis was the most common cause of death in infants 3–<7 days (50.9%, 54 of 106) and 7-<60 days (36.7%, 33 of 90).

#### Causes of deaths among registered infants

Overall, the mortality rate among registered infants in the verbal autopsy study was 25.7 per 1000 live births (1618 of 63,047; 67 deaths identified by CHWs were not included in the verbal autopsy study and four infants were excluded for whom age and cause of death were not specified) in the young infant period (days 0–<60) (figure 1; table 1). The cause could not be determined in 19.2% (311 of 1618) of infants, ranging from 9.9% (60 of 606) in infants 0–<3 days of age to 30.2% (222 of 736) in infants 7–<60 days. Approximately one-third (37.5%, 606 of 1618) occurred in infants 0–<3 days of age (online supplemental figure S2C). In infants 0–<3 days of age, infection/sepsis (35.0%, 212 of 606) and birth asphyxia (34.5%, 209 of 606) were the major causes of death, whereas preterm birth/LBW was identified as the cause in 15.4% (93 of 606) of deaths. Infection/sepsis was the predominant cause in infants 3–<7 days (56.9%, 157 of 276) and 7–<60 days (50.3%, 370 of 736) of age.

### Factors associated with mortality

#### Sample description

Online supplemental table S2 summarises the descriptive information on 63 114 registered infants and their mothers that were included in the risk factor analysis; stillbirths and unregistered infants were excluded from this analysis. Mother’s average age was 27.0 years (SD 5.6); 11.9% had experienced a prior neonatal death, 42.0% had no education, 81.3% attended any antenatal care, 20.0% and 0.9% sought care for pregnancy complications from a qualified or unqualified provider, respectively, and 54.4% gave birth in a health facility. Infants had a mean birth weight of 2737 g (SD 507), 51.4% were male, 18.7% were born preterm, 13.9% were resuscitated immediately after birth and 67.6% were fed colostrum immediately after birth.

#### Infants aged 0–<3 days

In the first 3 days, mortality from infection/sepsis (148 deaths in 42 506 infants) was most strongly associated with resuscitation of the baby (adjusted HR (aHR) 4.5, 95% CI 3.2 to 6.3] (table 2). Other factors associated with higher mortality attributed to infection/sepsis in the first 3 days were history of a prior neonatal death (aHR 1.6, 95% CI 1.1 to 2.4) and use of antibiotics during labour (aHR 1.5, 95% CI 1.04 to 2.2). Higher birth weight (aHR 0.13, 95% CI 0.1 to 0.2), birth in a health facility (aHR 0.4, 95% CI 0.3 to 0.6), female sex (aHR 0.6, 95% CI 0.4 to 0.8) and dry cord care (aHR 0.6, 95% CI 0.4 to 0.97) were associated with lower mortality from infection/sepsis.

**Table 2:** Factors associated with mortality in infants aged 0–<3 days

Variable	Category	Unadjusted Hazard Ratio (HR) (95% CI)	P value	Adjusted HR (95% CI)	P value
A. Infection/sepsis (n=148 deaths* among 42 506 registered infants)					
Antibiotics during labour	Yes	1.2 (0.9 to 1.6)	0.191	1.5 (1.04 to 2.2)	0.028
Baby resuscitated	Yes	4.3 (3.3 to 5.6)	<0.001	4.5 (3.2 to 6.3)	<0.001
Birth weight (per 1 kg increment)		0.14 (0.11 to 0.19)	<0.001	0.13 (0.1 to 0.2)	<0.001
Dry cord care	Yes	0.8 (0.6 to 1.1)	0.143	0.6 (0.4 to 0.97)	0.037
Place of birth	Health facility	0.4 (0.3 to 0.5)	<0.001	0.4 (0.3 to 0.6)	<0.001
Prior neonatal death	Yes	1.9 (1.4 to 2.7)	<0.001	1.6 (1.1 to 2.4)	0.016
Sex of baby (ref=boy)	Girl	0.6 (0.5 to 0.8)	<0.001	0.6 (0.4 to 0.8)	0.002
Ventilation for cooking (ref=needed but not available) (Overall p value=0.005)	Needed and available	1.2 (0.8 to 2.0)	0.385	1.8 (0.96 to 3.5)	0.067
	Not needed	0.9 (0.5 to 1.5)	0.586	1.0 (0.5 to 2.0)	0.932
B. Birth asphyxia (n=120 deaths† among 44 311 registered infants)					
Antibiotics during labour	Yes	2.5 (1.8 to 3.4)	<0.001	1.7 (1.1 to 2.5)	0.019
Baby resuscitated	Yes	19.0 (13.8 to 26.3)	<0.001	13.4 (8.7 to 20.7)	<0.001
Betel chewing	Yes	1.5 (1.1 to 2.1)	0.008	2.1 (1.3 to 3.4)	0.003
Birth weight (per 1 kg increment)	Yes	0.3 (0.2 to 0.34)	<0.001	0.3 (0.2 to 0.5)	<0.001
Congenital anomalies	Yes	6.2 (2.6 to 15.1)	<0.001	4.5 (1.6 to 12.4)	0.003
Dry cord care	Yes	0.7 (0.5 to 0.97)	0.027	0.6 (0.4 to 0.9)	0.009
Immediate breastfeeding	Yes	0.3 (0.2 to 0.4)	<0.001	0.3 (0.2 to 0.5)	<0.001
Wealth index		0.6 (0.5 to 0.7)	<0.001	0.7 (0.6 to 0.9)	0.006
C. Preterm birth/low birth weight (n=77 deaths‡ among 21 613 registered infants)					
Any antenatal care	Yes	0.5 (0.3 to 0.8)	0.002	0.6 (0.3 to 0.9)	0.026
Baby resuscitated	Yes	2.9 (1.9 to 4.7)	<0.001	2.9 (1.8 to 4.6)	<0.001
Dry cord care	Yes	0.5 (0.3 to 0.8)	0.006	0.4 (0.2 to 0.7)	0.001
Immediate breastfeeding	Yes	0.6 (0.4 to 0.9)	0.007	0.5 (0.3 to 0.7)	0.001
Multiple births	Yes	6.4 (3.8 to 10.5)	<0.001	7.0 (4.0 to 12.3)	<0.001
Qualified birth attendant	Yes	0.5 (0.4 to 0.8)	0.005	0.4 (0.2 to 0.7)	0.001

* 20 537 infants with 64 deaths were excluded due to missing values for risk factors.

† 18 732 infants with 89 deaths were excluded due to missing values for risk factors.

‡ 3554 infants with 13 deaths were excluded due to missing values for risk factors.

Deaths attributed to birth asphyxia (120 deaths in 44 311 infants) also had the strongest association with whether the baby was resuscitated (aHR 13.4, 95% CI 8.7 to 20.7), followed by reporting of congenital anomalies (aHR 4.5, 95% CI 1.6 to 12.4) (table 2). Other factors associated with higher mortality in the first 3 days after birth due to birth asphyxia were maternal betel chewing (aHR 2.1, 95% CI 1.3 to 3.4) and use of antibiotics during labour (aHR 1.7, 95% CI 1.1 to 2.5). Factors associated with lower hazard for mortality due to birth asphyxia included higher birth weight (aHR 0.3, 95% CI 0.2 to 0.5), immediate breastfeeding (aHR 0.3, 95% CI 0.2 to 0.5), dry cord care (aHR 0.6, 95% CI 0.4 to 0.9) and higher wealth (aHR 0.7, 95% CI 0.6 to 0.9).

Mortality attributed to preterm birth/LBW (77 deaths in 21 613 infants) was most strongly associated with multiple births (aHR 7.0, 95% CI 4.0 to 12.3) followed by whether the baby was resuscitated at birth (aHR 2.9, 95% CI 1.8 to 4.6) (table 2). Dry cord care (aHR 0.4, 95% CI 0.2 to 0.7), birth attendance by a qualified provider (aHR 0.4, 95% CI 0.2 to 0.7), immediate breastfeeding (aHR 0.5, 95% CI 0.3 to 0.7) and any antenatal care attendance (aHR 0.6, 95% CI 0.3 to 0.9) were associated with lower mortality due to preterm birth/LBW.

#### Infant aged 3–<7 days

Among infants 3–<7 days of age, mortality attributed to infection/sepsis (109 deaths in 46 535 infants) was most strongly associated with resuscitation at birth (aHR 2.6, 95% CI 1.7 to 3.9), followed closely by multiple births (aHR 2.5, 95% CI 1.3 to 4.7) and seeking care for labour complications from a qualified provider (aHR 2.2, 95% CI 1.2 to 3.8) (table 3). Higher birth weight (aHR 0.2, 95% CI 0.1 to 0.3), maternal education [primary (aHR 0.5, 95% CI 0.3 to 0.9), secondary (aHR 0.5, 95% CI 0.3 to 0.8), birth attendance by a qualified provider (aHR 0.4, 95% CI 0.3 to 0.6) and female sex (aHR 0.7, 95% CI 0.5 to 0.97) were associated with lower hazard of mortality from infection/sepsis.

**Table 3:** Factors associated with mortality in infants aged 3–<7 days

Variable	Category	Unadjusted Hazard Ratio (HR) (95% CI)	P value	Adjusted HR (95% CI)	P value
A. Infection/sepsis (n=109 deaths* among 46 535 registered infants)					
Baby resuscitated	Yes	2.6 (1.8 to 3.7)	<0.001	2.6 (1.7 to 3.9)	<0.001
Birth weight (per 1 kg increment)		0.2 (0.1 to 0.2)	<0.001	0.2 (0.1 to 0.3)	<0.001
Seeking care for labour complications (ref=no complications) (Overall p value=0.023)	Care from qualified provider	2.1 (1.4 to 3.2)	0.001	2.2 (1.2 to 3.8)	0.006
	Did not seek care from qualified provider	1.5 (0.5 to 4.7)	0.486	1.2 (0.4 to 3.8)	0.763
Mother’s highest education (ref=no education) (Overall p value=0.002)	Primary	0.6 (0.4 to 0.9)	0.011	0.5 (0.3 to 0.9)	0.014
	Secondary	0.3 (0.2 to 0.5)	<0.001	0.5 (0.3 to 0.8)	0.004
	College or higher	0.1 (0.02 to 0.5)	0.002	0.2 (0 to 1.1)	0.067
Multiple births	Yes	5.5 (3.2 to 9.3)	0	2.5 (1.3 to 4.7)	0.005
Qualified birth attendant	Yes	0.6 (0.4 to 0.8)	0.001	0.4 (0.3 to 0.6)	0
Sex of baby (ref=boy)	Girl	0.6 (0.5 to 0.9)	0.005	0.7 (0.5 to 0.97)	0.034
B. Birth asphyxia (n=28 deaths† among 45 209 registered infants)					
Baby resuscitated	Yes	11.6 (6.0 to 22.1)	<0.001	7.8 (3.5 to 17.1)	<0.001
Birth weight (per 1 kg increment)		0.2 (0.1 to 0.3)	<0.001	0.2 (0.1 to 0.4)	<0.001
Immediate breastfeeding	Yes	0.1 (0.1 to 0.2)	<0.001	0.2 (0.1 to 0.6)	<0.001
C. Preterm birth/low birth weight (n=19 deaths‡ among 17 585 registered infants)					
Immediate breastfeeding	Yes	0.2 (0.1 to 0.6)	0.002	0.1 (0.04 to 0.4)	<0.001
Multiple births	Yes	10.1 (4.2 to 24.4)	<0.001	8.4 (3.0 to 23.5)	<0.001

* 15 902 infants with 48 deaths were excluded due to missing values for risk factors.

† 17 228 infants with 14 deaths were excluded due to missing values for risk factors.

‡ 6864 infants with five deaths were excluded due to missing values for risk factors.

Mortality Attributed to asphyxia (28 deaths in 45 209 infants) was most strongly associated with resuscitation at birth (aHR 7.8, 95% CI 3.5 to 17.1) (table 3). Lower hazard of mortality was associated with higher birth weight (aHR 0.2, 95% CI 0.1 to 0.4) and immediate breastfeeding (aHR 0.2, 95% CI 0.1 to 0.6).

Multiple births had the strongest association with higher mortality from preterm birth/LBW (19 deaths in 17 853 infants) (aHR 8.4, 95% CI 3.0 to 23.5) (table 3). Immediate breastfeeding (aHR 0.1, 95% CI 0.04 to 0.4) was associated with lower mortality from preterm birth/LBW.

#### Infant aged 7–<60 days

In infants aged 7–<60 days, mortality from infection/sepsis (243 deaths in 43 054 infants) was most strongly associated with congenital anomalies (aHR 5.9, 95% CI 2.6 to 13.3) and multiple births (aHR 3.2, 95% CI 2.1 to 4.7) (table 4). Other factors associated with higher mortality from infection/sepsis were preterm birth (aHR 1.6, 95% CI 1.2 to 2.0), exposure to secondhand smoke (aHR 1.6, 95% CI 1.1 to 2.3), history of a prior neonatal death (aHR 1.5, 95% CI 1.1 to 2.0), history of resuscitation at birth (aHR 1.4, 95% CI 1.01 to 1.9) and risk of intra-amniotic infection (aHR 1.4, 95% CI 1.1 to 1.8). Lower hazard of mortality from infection/sepsis was associated with higher birth weight (aHR 0.2, 95% CI 0.2 to 0.3), birth in a health facility (aHR 0.7, 95% CI 0.5 to 0.9) and maternal education (secondary (aHR 0.6, 95% CI 0.4 to 0.8), college or higher (aHR 0.4, 95% CI 0.2 to 0.995)).

**Table 4:** Factors associated with mortality in infants aged 7–<60 days

Variable	Category	Unadjusted Hazard Ratio (HR) (95% CI)	P value	Adjusted HR (95% CI)	P value
A. Infection/sepsis (n=243 deaths* among 43 054 infants)					
Baby resuscitated	Yes	1.6 (1.3 to 2.1)	<0.001	1.4 (1.01 to 1.9)	0.041
Birth weight (per 1 kg increment)		0.2 (0.1 to 0.2)	<0.001	0.2 (0.2 to 0.3)	<0.001
Congenital anomalies	Yes	6.0 (3.0 to 12)	<0.001	5.9 (2.6 to 13.3)	<0.001
Intra-amniotic infection risk	Yes	1.4 (1.2 to 1.8)	<0.001	1.4 (1.1 to 1.8)	0.020
Mother’s highest education (ref=no education) (Overall p value=0.009)	Primary	0.7 (0.6 to 0.9)	0.014	0.8 (0.6 to 1.1)	0.178
	Secondary	0.4 (0.3 to 0.5)	<0.001	0.6 (0.4 to 0.8)	0.002
	College or higher	0.2 (0.1 to 0.5)	<0.001	0.4 (0.2 to 0.995)	0.049
Multiple births	Yes	7.0 (5.1 to 9.7)	<0.001	3.2 (2.1 to 4.7)	<0.001
Place of birth	Health facility	0.8 (0.6 to 0.95)	0.016	0.7 (0.5 to 0.9)	0.009
Preterm birth	Yes	2.4 (1.9 to 3.0)	<0.001	1.6 (1.2 to 2.0)	0.001
Prior neonatal death	Yes	2.0 (1.5 to 2.5)	<0.001	1.5 (1.1 to 2.0)	0.012
Exposure to secondhand smoke	Yes	1.8 (1.3 to 2.4)	<0.001	1.6 (1.1 to 2.3)	0.014
Ventilation for cooking (ref=needed but not available) (Overall p value=0.035)	Needed and available	1.1 (0.7 to 1.7)	0.652	1.1 (0.7 to 1.8)	0.61
	Not needed	2.0 (1.3 to 3.0)	0.001	1.6 (0.98 to 2.6)	0.061
B. Birth asphyxia (n=27 deaths† among 43 526 infants)					
Antibiotics during labour	Yes	7.7 (2.7 to 21.6)	<0.001	3.0 (1.01 to 8.8)	0.048
Baby resuscitated	Yes	8.6 (4.5 to 16.4)	<0.001	3.3 (1.5 to 7.1)	0.002
Birth weight (per 1 kg increment)		0.1 (0.1 to 0.2)	<0.001	0.2 (0.1 to 0.3)	<0.001
Immediate breastfeeding	Yes	0.1 (0.03 to 0.2)	<0.001	0.1 (0.02 to 0.2)	<0.001
Iron supplementation	Yes	0.6 (0.3 to 1.3)	0.195	3.7 (1.1 to 12.4)	0.037
Wealth index		0.7 (0.5 to 0.99)	0.041	0.6 (0.4 to 0.95)	0.030
C. Preterm birth/low birth weight (n=14 deaths‡ among 19 192 infants)					
Baby resuscitated	Yes	1.9 (0.7 to 4.7)	0.192	4.2 (1.4 to 12.3)	0.009
Seeking care for labour complications (ref=no complications) (Overall p value=0.012)	Care from a qualified provider	0.5 (0.1 to 3.5)	0.464	0.5 (0.1 to 4.1)	0.534
	Did not seek care from a qualified provider	6.1 (1.4 to 26.1)	0.015	12.2 (2.2 to 67.8)	0.004
Multiple births	Yes	2.0 (0.9 to 4.5)	0.109	5.9 (1.6 to 21.7)	0.007
New blade for umbilical cord	Yes	1.7 (0.8 to 3.6)	0.202	6.2 (1.6 to 23.8)	0.009
Place of birth in a health facility	Yes	1.9 (0.7 to 4.7)	0.192	4.2 (1.4 to 12.3)	0.009

* 19 105 infants with 127 deaths were excluded due to missing values for risk factors.

† 18 633 infants with 11 deaths were excluded due to missing values for risk factors.

‡ 5322 infants with 13 deaths were excluded due to missing values for risk factors.

Deaths attributed to birth asphyxia (27 deaths in 43 526 infants) were associated with resuscitation at birth (aHR 3.3, 95% CI 1.5 to 7.1), antibiotic use during labour (aHR 3.0, 95% CI 1.01 to 8.8) and reports of iron supplementation (aHR 3.7, 95% CI 1.1 to 12.4) (table 4). Lower hazard of mortality was associated with immediate breastfeeding (aHR 0.1, 95% CI 0.02 to 0.2), higher birth weight (aHR 0.2, 95% CI 0.1 to 0.3) and higher wealth (aHR 0.6, 95% CI 0.4 to 0.95).

Among infants born preterm/LBW (14 deaths in 19 192 infants), lack of care-seeking for labour complications from a qualified provider (aHR 12.2, 95% CI 2.2 to 67.8), use of a new blade to cut the umbilical cord (aHR 6.2, 95% CI 1.6 to 23.8), multiple births (aHR 5.9, 95% CI 1.6 to 21.7), birth in a health facility (aHR 4.2, 95% CI 1.4 to 12.3) and history of resuscitation at birth (aHR 4.2, 95% CI 1.4 to 12.3) were associated with higher mortality attributed to preterm/LBW (table 4).

## Discussion

This population-based, observational study of 73 622 infants across five sites in three countries of South Asia identified 4638 deaths in the first 60 days after birth. Deaths were roughly divided into one-third stillbirths, one-third infants who died early before they could be registered into the study and one-third infants who were registered into the study and subsequently died in the young infant period. The study sites were characterised by poverty, low maternal education, high rates of antenatal care but poor care-seeking for complications and high mortality rates (31.5 stillbirths per 1000 births, 43.0 young infant deaths per 1000 live births and 60.6 perinatal deaths per 1000 live births).

Considering the entire cohort of infants (unregistered and registered), nearly 60% of deaths over the young infant period (days 0–<60) occurred in the first 3 days after birth and nearly three-fourths (72.1%) occurred in the first week. Approximately 90% of young infant deaths attributed to birth asphyxia and nearly 80% of deaths attributed to preterm birth/LBW took place in the first 3 days; in comparison, approximately one-third of deaths due to infection/sepsis and half of deaths due to congenital malformations occurred in the first 3 days. Over the entire young infant period, deaths were attributed most commonly to infection/sepsis (32.5% of all deaths) and birth asphyxia (29.0%) with preterm birth a distant third (14.1%). Birth asphyxia assumed even greater prominence (43.1%) among early deaths in the first 3 days, whereas infection/sepsis was most prominent in causing half (50.8%) of deaths over the remainder of the young infant period (days 3-<60).

It was instructive to further examine patterns of death in unregistered and registered infants. Our study was designed to identify the community-based aetiology of infections and emphasised early and population-based ascertainment and reach to enable registration of all liveborn infants as soon as possible after birth. Thus, deaths of the unregistered infants occurred early after birth (85.4% within 3 days of birth) before CHWs could reach the household and are more readily missed in less intensive surveillance efforts outside such a research context. Data sources included in global databases used to model mortality rates and causes include civil registration and vital statistics systems, national health management systems, birth registries and relevant published studies such as cross-sectional studies, hospital data, research trials or programme data.^4^,^734^ Data are relatively scarce from low-resource settings and will tend to more closely resemble that of our registered infants who were identified during routine household visits by CHWs and will miss the additional information—in our case, from half of deaths—that could be gleaned through intensive community and household surveillance to identify infants like our unregistered infants, with the additional advantage that these population-based data have minimal risk for selection and recall bias. As expected, the proportion of early deaths in days 0–<3 varied markedly between unregistered (85.4%) and registered infants (37.4%), demonstrating the importance of complete demographic surveillance for capturing early deaths. In addition to differences in the timing of death, the distribution of causes of death differed between unregistered and registered infants, most notably in the higher proportion of all young infant deaths due to birth asphyxia in unregistered (42.3%) compared with registered (17.8%) infants. For infection/sepsis, the opposite relationship was found, with 16.6% of deaths attributed to infection/sepsis in unregistered infants compared with 45.6% in registered infants. These data suggest that modelling based on global datasets may tend to underappreciate the importance of birth asphyxia while overcounting deaths due to infection.

The rates of stillbirths and young infant deaths found here for community-based sites in Bangladesh, India and Pakistan were similar to the rates reported for the same three countries in the population-based AMANHI study (stillbirths: 35·1 per 1000 births; neonatal deaths: 43·0 per 1000 livebirths)^16^ and are higher than recent modelled estimates for South Asia.^3 5 35^ The ratio of 1.2 young infant deaths to each stillbirth in our sites was similar to that found in AMANHI for the ratio of neonatal deaths to stillbirths (1.1), with our higher ratio due in part to the relatively small numbers of deaths that occurred beyond the neonatal period in the second month. Moreover, distributions of death by age and cause were also similar to those reported for the AMANHI study. While we found that 59.2% and 72.1% of young infant deaths occurred in the first 3 and 7 days after birth, AMANHI reported that 44% of neonatal deaths occurred in labour and up to 24 hours after birth and 80% occurred in the first 7 days. These population-based rates of early deaths are slightly higher than or similar to those reported in studies modelling the global burden of neonatal deaths.^36^ Of particular note, our topmost causes of young infant deaths (infections/sepsis, 32.5%; birth asphyxia, 29.0%) were similar to AMANHI causes of neonatal deaths (birth asphyxia, 40%; severe infections, 35%), and both studies found lower proportions of deaths attributed to preterm birth (our study, 14.1%, AMANHI, 19%), contrasting with modelled global data which report that the top cause of neonatal deaths is complications of preterm birth.^4 8^ A global review focused on community-based data highlighted the importance of infections as the cause of 8–80% of neonatal deaths and as many as 42% of deaths in the first week after birth.^37^

Our study extends analysis of neonatal deaths beyond that reported by AMANHI to examine risk factors for mortality. We limited this analysis to registered infants (n=63 114) for whom we had complete verbal autopsy outcome and age-of-death data from CHWs’ interviews at household level (n=63 043). In the first 3 days after birth, the factor overall most strongly associated with risk for mortality due to infection/sepsis or birth asphyxia was need for resuscitation of the infant at birth, which was also the factor with the second strongest association with death due to preterm birth/LBW. Resuscitation as a risk factor for early neonatal mortality may reflect not only the presence of birth asphyxia but likely also the presence of serious illness due to other causes with a common clinical presentation at the time of birth. Use of antibiotics during labour (perhaps in response to preterm premature rupture of membranes) was also an important risk factor in the first 3 days for death due to infection/sepsis or birth asphyxia. Christian *et al* showed previously that the combined presence of signs of birth asphyxia and infection in the early postnatal period was associated with particularly elevated risk for infant mortality.^38^ Bang *et al* similarly emphasised the importance of addressing both birth asphyxia and infections to reduce neonatal mortality in rural India.^39^ Poor quality of implementation may also have been a factor in the early deaths of infants in our study who received resuscitation and intrapartum antibiotics. Congenital anomaly was an important risk factor for death due to birth asphyxia for unknown reasons but may have complicated resuscitation efforts. This analysis thus highlights the importance of quality care during the antenatal and intrapartum periods. Immediate breastfeeding and cord care were particularly important factors associated with reduced risk for early death, highlighting the critical role of early essential newborn care.^40^ Higher birth weight was also protective for death due to infection/sepsis, as reported previously,^20^ and from birth asphyxia. Facility birth (associated with protection from death due to infection/sepsis), level of wealth (protection from death due to birth asphyxia) and antenatal care attendance and use of a qualified birth attendant (protection from death due to preterm birth) may reflect access to care.^20^ Similar factors were among the most important risk and protective factors found for infants who died in the second half of the first week, with the addition of maternal education as a protective factor from death due to infections/sepsis, possibly associated with better access to care and improved hygiene practices,^41^ although cord care was not identified as a risk factor for death due to infections/sepsis in this age group. Female sex was also protective in the first week after birth from death due to infections, likely reflecting their early biological advantage over males and consistent with the previously reported finding that male sex was a risk factor for community acquisition of serious bacterial infection.^20 42 43^ Our finding of seeking care for labour complications from a qualified provider as a risk factor for death due to infection/sepsis during the second half of the first week may reflect poor quality of care. Our findings are consistent with other data from South Asia showing that low gestational age, male sex and preterm premature rupture of membranes are important risk factors for neonatal sepsis and that neonatal death is strongly associated with late antepartum maternal infection.^44^,^46^

After the first week, many of the same risk and protective factors were present. Many risk factors for deaths attributed to infection/sepsis in infants 7–<60 days of age appeared to reflect exposure or impaired defence (ie, congenital anomalies, multiple/preterm birth, smoke exposure), existing morbidity and/or poor quality of care at birth (eg, resuscitation, risk of intra-amniotic infection at birth). Facility birth and maternal education were protective from death due to infection/sepsis, perhaps reflective of access to care. Deaths due to birth asphyxia may be related to underlying infection (intrapartum antibiotic use),^38^ while immediate breastfeeding was protective, as for deaths in the first week, as was level of wealth, which like education may facilitate access to care. The role of iron supplementation as a risk factor for mortality due to birth asphyxia may reflect the presence of maternal anaemia in a context of poor implementation of supplementation.^47^ Deaths attributed to prematurity/LBW after the first week appear to reflect a combination of underlying risk (need for resuscitation, multiple birth) and quality of care received (lack of care seeking from a qualified provider for labour complications). The interpretation of the use of a new blade for umbilical cord cutting as a risk factor is not clear.

Strengths of our study include its population base (including intensive CHW home visits) and large sample size, designed to maximise the capture of births early as well as outcomes for all pregnancies and the harmonised study design, training, implementation, data collection and analysis across sites, including standardised, rigorous assignment of causes of essentially all deaths, enabling us to pool individual-level data across sites. Enrolment occurred over 18–27 months, minimising seasonal effects. Moreover, this observational study was not linked to studies of interventions which could alter rates and/or distributions of causes of mortality. This study extends knowledge beyond modelled estimates which advance prematurity as the top cause of young infant deaths globally, including in sub-Saharan Africa and South Asia; in agreement with the AMANHI study, we find that infections/sepsis and birth asphyxia are the two topmost causes of mortality, followed by preterm birth as the third most important cause. Beyond the analysis conducted by the AMANHI group,^16^ we further break down causes of mortality by unregistered and registered infants, showing that a preponderance of deaths in the unregistered infants occurred early and were due to birth asphyxia and infections/sepsis. These data serve to further reinforce the finding that study designs that lack intensive population-based surveillance for pregnancies and vital status of young infants will tend to underestimate birth asphyxia and infections as causes of deaths. Moreover, our study extends knowledge beyond the AMANHI study by examining risk factors for each of the major causes of neonatal death. Our study is the largest population-based study that we are aware of that has examined risk factors for causes of young infant deaths; furthermore, we break this down into age subgroups to emphasise the differences in causes and risk factors in very early deaths as opposed to those that occur after the first week following birth. Potential weaknesses include the lack of data on management of infants identified with pSBIs, the limited number of variables available for analysis of risk factors across four causes of mortality and, in some cases, recall bias associated with delayed administration of verbal autopsy.^23^ Despite careful surveillance for pregnancies and vital status outcomes, it is possible that in our study some pregnancies were missed and some deaths were misclassified between stillbirth and neonatal death. We also note that the analysis of risk factors for mortality is based on data from registered infants; despite our systematic efforts to reach all infants born into our study populations immediately after birth and before death, we nevertheless were unable to register a substantial number of infants who died early after birth. We also note that we could not identify a cause of death based on the data provided in the verbal autopsy for nearly 14% of deaths, which may lead to bias in the proportions of causes of death which are reported. However, verbal autopsy studies typically miss a similar to higher proportion of deaths.

Our data reinforce the need to intervene early, in the antenatal, intrapartum and immediate postpartum periods (eg, the first 3 days) to avert stillbirths and early infant deaths, particularly due to birth asphyxia and preterm birth, but also due to infections and congenital anomalies for which half of young infant deaths occur in the first week.^2^ Globally, there is a shift in emphasis in neonatal health programming and research toward facility-based care, childbirth care and care for small and sick or small vulnerable newborns, including preterm infants.^48^,^50^ It is important for programmes in high mortality settings in South Asia to continue to focus on addressing birth asphyxia and infections as primary causes of death at population level, while recognising that preventing and managing preterm birth and congenital anomalies also require concerted attention, especially given trends showing that the proportions of neonatal deaths due to these causes are rising.^3 7^

## Supplementary material

10.1136/bmjgh-2024-018433 online supplemental file 1

## Data Availability

Data are available upon reasonable request.
