# Supplementary material for: Causes and risk factors for deaths in young infants in South Asia: the ANISA prospective population-based observational cohort study
Source: BMJ Glob Health. 2025 Nov 3;10(11):e018433. doi: 10.1136/bmjgh-2024-018433 (PMC12584570; doi:10.1136/bmjgh-2024-018433)

**Causes and risk factors for deaths in young infants in South Asia: the ANISA prospective**

**population-based observational cohort study**

**Supplemental Tables and Figures**

**Supplemental table S1. Potential risk factors for causes of death of young infants 0-<59 days of age in five study sites in**

**India, Bangladesh and Pakistan in the Aetiology of Neonatal Infection in South Asia (ANISA) study**

| **Variable** | **Description** |
| --- | --- |
| **Reproductive history and current pregnancy** | |
| History of baby death | Ever given birth to a boy or a girl who was born alive but later died before completing 1 month |
| **Mother’s nutrition** | |
| Mother’s nutritional status during pregnancy | Mid-upper arm circumference in cm |
| **Socio-demographics** |  |
| Mother’s age | Age in years |
| Mother’s education | The highest level of school attended |
| Mother’s employment | The mother does not work for income generation, the mother works at home only for income generation, or the mother works outside home for income generation. |
| Mother’s agency | Whether the mother is involved alone or jointly with her husband or someone else in making decisions about:   - Child healthcare - Other matters - Not involved in any decisions |
| Mother’s mobility | - Mother can go alone to the health center or hospital - Not able to go alone or having to be accompanied by someone |
| Household wealth | Wealth index is constructed from the following household assets and characteristics:   - Improved main roofing material: natural roofing (no roof, thatch/palm leaf/reed/grass, mud, sod/mud and grass mixture, wood, rudimentary roofing, rustic mat, bamboo, raw wood planks/ timber/ cardboard, plastic/polyethylene sheeting, unburnt brick, loosely packed stone)   Versus  Finished roofing (metal/GI or iron sheet/tin, finished wood, calamine/cement fiber, asbestos sheet, reinforced, brick cement/ RCC/ concrete/cement, roofing shingles, tiles, slate, burnt brick, tent)   - Improved main floor material: finished floor (parquet/polished wood, Vinyl/asphalt, ceramic tiles, cement, chips/terrazzo/mosaic, carpet/mat, polished stone/marble/granite)   Versus  Natural floor (mud/clay/earth/sand, dung), or rudimentary floor (raw wood planks, palm/bamboo, brick with no lime/cement, rough stone with no lime/cement)   - Improved main wall material: finished wall (cement/concrete, stone with lime/cement, burnt bricks with lime/cement, finished wood planks/shingles, cement blocks, or metal sheet)   Versus  None, natural wall (none, cane/palm/trunk/bamboo, dirt/mud, stone, grass/reed/thatch/sticks), or rudimentary wall (bamboo with mud, stone with mud, plywood, cardboard/plastic, sunburn brick, raw wood/reused wood)   - Cooking stove and fuel: LPG or biogas stove, electric stove, kerosene stove   Versus  Traditional or improved mud stove with biomass, charcoal, or animal residue   - Overcrowding (number of household members per sleeping room > 5) - Improved main source of drinking water: piped, tube well/hand pump, rainwater, filter water   Versus  Dug well or spring water, water tanker/cart, surface, bottled, or from neighbors   - An improved type of toilet facility: flushed to piped sewer system, septic tank, pit latrine, or elsewhere, ventilated improved pit/biogas latrine, pit latrine with slab, twin pit/composting toilet or dry toilet   Versus  Pit latrine without a slab, bucket toilet, hanging toilet or no facility/bush/open space or field   - Electricity - Water pump - CD/DVD player - Black and white television - Color television - Refrigerator - Radio/transistor - Mobile telephone - Land telephone - Computer - Bicycle - Tractor/boat with motor - Car/truck - Motorcycle/scooter - Rickshaw/van - Almirah/cabinet |
| **Antenatal care (Mother data file)** | |
| Medical check-ups during pregnancy | Received any medical check-up during pregnancy |
| Number of antenatal care (ANC) visits during pregnancy | Number of ANC visits to a qualified provider during pregnancy |
| Tetanus immunization in the current pregnancy | Received two or more tetanus toxoid doses in the current pregnancy |
| Iron supplementation | Taken any iron tablet/syrup during pregnancy |
| Cigarette smoking during the current pregnancy | Ever smoked cigarettes during the current pregnancy |
| Hookah smoking during the current pregnancy | Ever smoked hookah during the current pregnancy |
| Tobacco chewing during the current pregnancy | Ever chewed tobacco during the current pregnancy |
| Batel leaf chewing during the current pregnancy | Ever chewed betel leaf during the current pregnancy |
| Exposure to second-hand smoke | Any person in the household smoked cigarettes while the mother was pregnant |
| Ventilation | Type of ventilation for stove if an indoor stove |
| Heavy physical work | Whether the mother performed heavy physical work or not |
| **Complications during pregnancy and health-seeking behavior** | |
| Experience of complications during the current pregnancy | - Mother had any of the following during pregnancy: high-grade fever, excessive bleeding, convulsions, swelling of the hands and feet, smelly discharge or other complications and sought care from a qualified provider (doctor, nurse, midwife or paramedic) - Mother had any of the following during pregnancy: high-grade fever, excessive bleeding, convulsions, swelling of the hands and feet, smelly discharge or other complications but did not seek care from a qualified provider - Mother had none of these complications |
| **Labor outcomes** | |
| Term birth | Whether delivery took place at or after 37 weeks |
| Multiple births | Whether the mother had a single baby or more than one baby |
| Sex | Whether the baby was male or female |
| Place of birth | Whether the birth took place in a health facility versus at home/on the way to the hospital |
| Birth attendant | Whether delivered by a qualified provider (doctor, nurse, midwife, paramedic) versus other provider |
| Risk of intra-amniotic infection | Presence of any of the following: water broke before the onset of labor pain, the non-clear color of amniotic fluid, or smelly vaginal discharge during labor |
| Use of antibiotics during labor | Whether the mother took antibiotics during labor. |
| Experience complications during labor | - Mother had any of the following during labor: excessive bleeding, prolonged labor, fetal malpresentation, convulsions, or retained placenta and received care from a qualified provider (doctor, nurse, midwife, or paramedic) - Mother had any of the following during labor: excessive bleeding, prolonged labor, fetal malpresentation, convulsions, or retained placenta but did not receive care from a qualified provider - Mother had none of those complications |
| Mother illness before delivery | Whether mother had a respiratory illness, skin pustules, or diarrhea within the 7 days preceding delivery |
| Household member illness before delivery | Whether any other member of the household had a respiratory illness, skin pustules, or diarrhea within the 7 days preceding delivery |
| **Postnatal care** |  |
| Resuscitation | Whether supportive measures for breathing were taken after the baby’s birth or not |
| Handler washed hands | Whether the person who handled the baby after birth washed their hands |
| Wiped immediately after birth | Timing for wiping baby just after birth = 0 vs > 0 minutes |
| Clean cloth for wiping | Whether the baby was wiped dry by a clean cloth |
| Wrapped immediately after birth | Timing of wrapping just after birth = 0 vs > 0 minutes |
| Clean cloth for wrapping | Whether the baby was wrapped in a clean cloth |
| The instrument used to cut the cord | The cord was cut with a new blade from the delivery kit or another new blade used blade/knife, scissors, bamboo strips, paper, cutter, tongs, cord not cut, other |
| Dry cord care | Whether anything was applied to the umbilical stump or not |
| Immediate breastfeeding | Whether the baby received colostrum immediately after birth or not |
| Birth weight | Newborn’s weight in kg |
| Congenital anomalies | Whether the mother reported congenital anomalies |

**Supplemental table S2. Distribution of potential factors associated with mortality, from mother-reported data collected**

**during pregnancy or the first home visit (total sample: N = 63,114 registered infants and their mothers)**

| **Continuous variables** | **N** | **Mean (SD)** |
| --- | --- | --- |
| Mother’s age (years) | 62,631 | 27.0 (5.6) |
| Mother’s arm circumference (cm) | 62,631 | 33.4 (24.6) (24.6)27.1 (5.6) |
| Infant birth weight (g) | 61,569 | 2,737 (507) |
| Household wealth index | 62,368 | -0.01 (1.0) |
| **Categorical variables** | **Frequency** | **Percent** |
|  |  |  |
| ***Maternal/antenatal*** |  |  |
| Mother’s work for income (cash or kind) |  |  |
|  |  |  |
| None | 51,964 | 82.3 |
| Outside the home | 7,562 | 12.0 |
| Inside the home | 3,105 | 4.9 |
| Missing | 483 | 0.8 |
| Decision-making involvement of the mother |  |  |
|  |  |  |
| None | 39,830 | 63.1 |
| In child healthcare | 21,516 | 34.1 |
| In other family decisions | 1,768 | 2.8 |
| Mother’s highest education |  |  |
|  |  |  |
| No education | 26,474 | 42.0 |
| Primary education | 12,412 | 19.7 |
| Secondary education | 17,264 | 27.4 |
| College or higher | 4,569 | 7.2 |
| Missing (includes only Madrasha) | 2,395 | 3.8 |
| Prior neonatal death (baby born alive but died later within one month) |  |  |
| Yes | 7,533 | 11.9 |
| No | 55,092 | 87.3 |
| Missing | 489 | 0.8 |
| Any antenatal care during pregnancy with index child |  |  |
|  |  |  |
| Yes | 51,303 | 81.3 |
| No | 11,809 | 18.7 |
| Missing | 2 | 0.00 |
| Two tetanus doses during pregnancy with index child |  |  |
|  |  |  |
| Yes | 29,227 | 46.3 |
| No | 33,883 | 53.7 |
| Missing | 4 | 0.01 |
| Iron supplementation during pregnancy with index child |  |  |
|  |  |  |
| Yes | 51,608 | 81.8 |
| No | 11,504 | 18.2 |
| Missing | 2 | 0.00 |
| Cigarette smoking |  |  |
|  |  |  |
| Yes | 539 | 0.9 |
| No | 62,573 | 99.1 |
| Missing | 2 | 0.00 |
| Mother used hookah |  |  |
|  |  |  |
| Yes | 346 | 0.6 |
| No | 62,766 | 99.5 |
| Missing | 2 | 0.00 |
| Mother chewed tobacco |  |  |
|  |  |  |
| Yes | 5,255 | 8.3 |
| No | 57,857 | 91.7 |
| Missing | 2 | 0.00 |
| Mother chewed betel |  |  |
|  |  |  |
| Yes | 12,076 | 19.1 |
| No | 51,036 | 80.9 |
| Missing | 2 | 0.00 |
| Mother’s exposure to second-hand smoke |  |  |
|  |  |  |
| Yes | 20,411 | 32.3 |
| No | 42,701 | 67.7 |
| Missing | 2 | 0.00 |
| Ventilation for cooking |  |  |
|  |  |  |
| Needed (cooks indoors) and available | 39,114 | 62.0 |
| Not needed (cooks outdoors or in veranda) | 18,048 | 28.6 |
| Needed but not available | 5,944 | 9.4 |
| Missing | 8 | 0.01 |
| Mother did heavy physical work during pregnancy |  |  |
|  |  |  |
| Yes | 14,090 | 22.3 |
| No | 49,016 | 77.7 |
| Missing | 8 | 0.01 |
| Seeking care for pregnancy complications |  |  |
|  |  |  |
| Sought care from a qualified provider (doctor, nurse, midwife or paramedic) | 12,639 | 20.0 |
| Sought care from a non-qualified provider (any other provider) | 571 | 0.9 |
| No pregnancy complications | 43,986 | 69.7 |
| Missing | 5,918 | 9.4 |
| Mother had a respiratory illness, skin pustules, or diarrhoea within 7 days before delivery |  |  |
|  |  |  |
| Yes | 12,849 | 20.4 |
| No | 50,159 | 79.5 |
| Missing | 106 | 0.2 |
| Household member had a respiratory illness before delivery |  |  |
|  |  |  |
| Yes | 4,606 | 7.3 |
| No | 58,508 | 92.7 |
| ***Intrapartum*** |  |  |
| Intra-amniotic infection risk (water broke before labor, amniotic fluid not clear, foul-smelling vaginal discharge during labor/delivery) |  |  |
|  |  |  |
| Yes | 21,566 | 34.2 |
| No | 41,546 | 65.8 |
| Missing | 2 | 0.00 |
| Antibiotics were given during labour |  |  |
|  |  |  |
| Yes | 32,043 | 50.8 |
| No | 28,812 | 45.7 |
| Missing | 2,259 | 3.6 |
| Delivered in a health facility |  |  |
|  |  |  |
| Yes | 34,352 | 54.4 |
| No (home or on the way to the hospital) | 28,743 | 45.5 |
| Missing | 19 | 0.03 |
| Delivery attended by a qualified birth attendant (doctor, nurse, midwife or paramedic) |  |  |
|  |  |  |
| Yes | 35,140 | 55.7 |
| No | 27,973 | 44.3 |
| Missing | 1 | 0.00 |
| Seeking care for labour complications |  |  |
| No labour complications | 52,748 | 83.6 |
| At least one complication: sought care from a qualified provider | 5,416 | 8.6 |
| At least one complication: did not seek care from a qualified provider | 909 | 1.4 |
| Missing | 4,041 | 6.4 |
| ***Infant/postpartum*** |  |  |
| Baby’s sex |  |  |
|  |  |  |
| Male | 32,419 | 51.4 |
| Female | 30,695 | 48.6 |
| Multiple births |  |  |
|  |  |  |
| Yes | 1270 | 2.0 |
| No | 61,844 | 98.0 |
| Preterm birth (before 37 weeks, calculated from the date of last menstrual period and date of delivery) |  |  |
|  |  |  |
| Yes | 11,811 | 18.7 |
| No | 48,068 | 76.2 |
| Missing | 3,235 | 5.1 |
| Baby resuscitated immediately after birth |  |  |
|  |  |  |
| Yes | 8,773 | 13.9 |
| No | 52,723 | 83.5 |
| Missing | 1,618 | 2.6 |
| Birth attendant washed hands |  |  |
|  |  |  |
| Yes | 55,405 | 87.8 |
| No | 4,192 | 6.6 |
| Missing | 3,517 | 5.6 |
| Baby wiped immediately after birth |  |  |
|  |  |  |
| Yes | 44,844 | 71.1 |
| No | 18,270 | 29.0 |
| New cloth used to wipe baby |  |  |
|  |  |  |
| Yes | 11,443 | 18.1 |
| No | 51,671 | 81.9 |
| Baby wrapped immediately after birth |  |  |
|  |  |  |
| Yes | 35,812 | 56.7 |
| No | 27,302 | 43.3 |
| New cloth used to wrap baby |  |  |
|  |  |  |
| Yes | 12,654 | 20.1 |
| No | 50,460 | 80.0 |
| New blade used to cut the cord |  |  |
|  |  |  |
| Yes | 32,332 | 51.2 |
| No | 30,782 | 48.8 |
| Dry cord care (nothing applied to the cord stump) |  |  |
|  |  |  |
| Yes | 43,954 | 69.6 |
| No | 17,575 | 27.9 |
| Missing | 1,585 | 2.5 |
| Immediate breastfeeding (given colostrum immediately after birth) |  |  |
|  |  |  |
| Yes | 42,669 | 67.6 |
| No | 20,197 | 32.0 |
| Missing | 248 | 0.4 |
| Congenital abnormalities |  |  |
|  |  |  |
| Yes | 262 | 0.4 |
| No | 62,512 | 99.1 |
| Missing | 340 | 0.5 |

**Supplemental figure S2A. Cumulative mortality of young infants in the Aetiology of Neonatal Infection**

**in South Asia (ANISA) study by cause of death, including all infants (unregistered and registered) except**

**stillbirths (n=2,320) and infants (n=4) who died for whom age-at-death and cause-of-death information**

**was not specified (N = 2,965 deaths among 71,200 infants)**


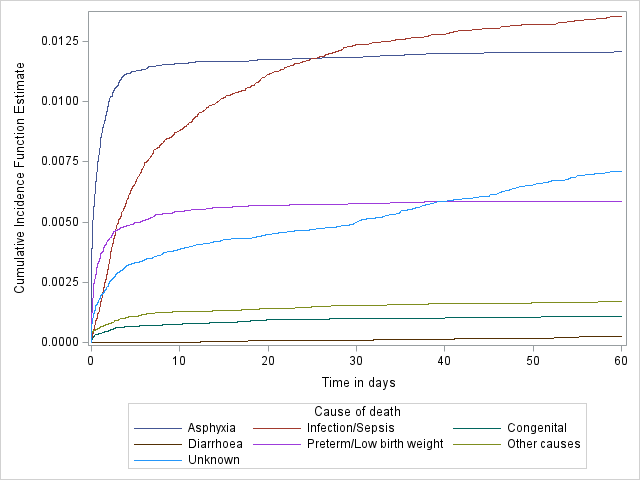


**Supplemental figure S2B. Cumulative mortality of unregistered infants by cause of death**

**(N = 1,347 deaths among 8,157 infants)**


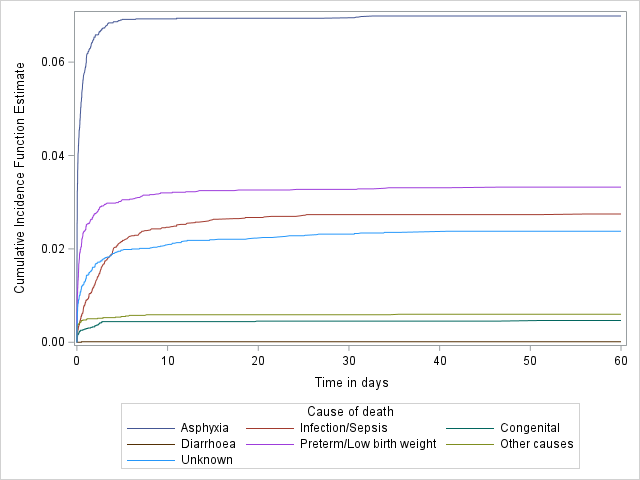


**Supplemental figure S2C. Cumulative mortality of registered infants in the Aetiology of Neonatal Infection**

**in South Asia (ANISA) study by cause of death (N = 1,618 deaths among 63,043 infants, excluding 4 infants**

**with no age at death or cause of death information)**


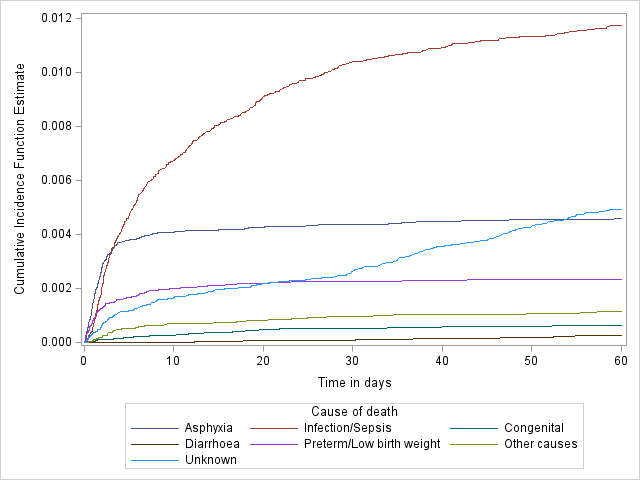

Supplement: online supplemental file 1 [file bmjgh-10-11-s001.docx]
